# Mapping the Influence of Artificial Intelligence in Prosthodontics: A Scoping Review of the Current Trends and Challenges

**DOI:** 10.3390/dj14090570

**Published:** 2026-09-06

**Authors:** Daiana Andrea Boca, Codruta-Eliza Ille, Anca Jivanescu

**Affiliations:** 1 Department of Prosthodontics, Faculty of Dental Medicine, “Victor Babes” University of Medicine and Pharmacy, 300580 Timișoara, Romania; daiana.boca@umft.ro (D.A.B.); jivanescu.anca@umft.ro (A.J.); 2 TADERP Research Center—Advanced and Digital Techniques for Endodontic, Restorative and Prosthetic Treatment, “Victor Babeș” University of Medicine and Pharmacy, 300041 Timişoara, Romania

**Keywords:** artificial intelligence, prosthodontics, machine learning, deep learning, digital workflow, CAD/CAM, scoping review

## Abstract

**Background/Objectives:** Artificial intelligence (AI) is transforming prosthodontic procedures into data-driven support systems. This scoping review systematically maps the literature using an evidence maturity framework, distinguishing it from recent applications-focused compilations by Aljulayfi, Schwendicke, and others. **Methods:** A comprehensive search of PubMed, Scopus, and Web of Science (January 2023–March 2026) was conducted in accordance with PRISMA-ScR guidelines, supplemented by targeted manual citation tracking. Fifty studies were included in the review. **Results:** The evidence base comprises approximately half primary research (computational, in vitro, clinical) and half review articles. Foundational diagnostic CNNs report high technical performance metrics (i.e., sensitivity 98.67%, precision 78.12%), while generative CAD/CAM approaches reduce design time by up to 52% in vitro. However, the evidence synthesis reveals a significant gap between algorithmic efficacy and clinical utility. Most applications currently reside at the “technical validation” stage, while emerging domains such as large language models remain largely experimental. **Conclusions:** Impressive computational metrics do not automatically equate to patient-centered clinical benefits. Current AI systems must be regarded as adjunctive decision-support instruments rather than autonomous tools. Future research must prioritize prospective multicenter validation, implementation science, explainability, and robust regulatory oversight.

## 1. Introduction

Artificial intelligence (AI) refers to computational systems capable of performing tasks that typically require human intelligence, including learning from experience, pattern recognition, problem solving, natural language understanding, and decision-making [1,2]. AI is a broad discipline comprising several subfields, including machine learning (ML), deep learning (DL), natural language processing, computer vision, and robotics [1,3]. ML uses algorithms to identify relationships within structured data and generate data-driven predictions or automated clinical decision support [4].

Traditional ML techniques rely on human-defined features before model training. Because feature selection is guided by domain expertise, these models may be relatively interpretable and computationally efficient [1,2]. DL, by contrast, is a subfield of ML that uses multilayer artificial neural networks (ANNs) to learn complex hierarchical patterns from high-dimensional data, such as clinical images [1]. DL is particularly effective for processing large datasets and now dominates image-based dental applications, although it requires substantial computational resources [3,4]. Common DL architectures include convolutional neural networks (CNNs) for image analysis and generative adversarial networks (GANs) for synthetic data generation and augmentation [1,5,6].

The integration of AI into prosthodontic workflows addresses several clinical challenges, including margin line detection, component selection, and the fabrication of fixed and removable prostheses [7,8,9]. Recent evidence suggests that AI-based systems can improve diagnostic accuracy and support clinicians in identifying appropriate intervention areas more efficiently [10,11]. Beyond diagnosis, these technologies may facilitate earlier disease detection, optimize workflow efficiency, and support patient-specific care and treatment planning [3,11].

AI has been utilized in many dental disciplines. However, many recent reviews have previously outlined basic AI applications in restorative dentistry and CAD/CAM processes, e.g., by Aljulayfi, Dawood, Schwendicke, and Rokhshad [5,6,7,8]. A comprehensive appraisal of the discipline demands going beyond descriptive catalogues of high-performing algorithms. The real difficulty in modern digital dentistry is not only to achieve great algorithmic accuracy in the lab but to effectively integrate these technologies into complicated and dynamic clinical contexts.

Thus, this scoping review attempts to map and synthesize existing uses of AI in prosthodontics, with a specific conceptual lens: to explore the trajectory of AI from isolated algorithmic advances to interconnected digital ecosystems. This study aims to analyze the literature through an evidence maturity framework to make a clear distinction between experimental promise, technical validation and clinical readiness, and to identify the translational impediments to routine use.

## 2. Materials and Methods

### 2.1. Study Design

The scoping review was performed in line with the PRISMA Extension for Scoping Reviews (PRISMA-ScR) [12] to facilitate methodological transparency using a standardized checklist and flow diagram.

Although a formal study protocol was not prospectively registered (e.g., in PROSPERO)—which is recognized as a methodological restriction—the study objectives, methods, and selection criteria were rigorously established prior to the literature search. This approach was implemented to support reproducible evidence mapping, ensure methodological transparency, and effectively mitigate the risk of post hoc reporting bias. The finalized PRISMA-ScR checklist is included in the Supplementary File S1.

### 2.2. Eligibility Criteria

Inclusion criteria for studies were: (1) studies investigating applications of artificial intelligence (AI), machine learning, deep learning, automation or robotics in dentistry; (2) studies related to prosthodontics or digital workflows (digital smile design, intraoral scanning, digital impressions, CAD/CAM systems, margin line detection, crown design, color/shade detection, robotics, implant dentistry); (3) original research articles, systematic reviews, scoping reviews, and narrative reviews. Narrative reviews were explicitly included to map bioethical considerations, educational applications, and broad implementation challenges where quantitative primary data remains scarce; (4) published in English; and (5) published between 1 January 2023 and 4 March 2026. This restricted publication period intentionally focuses on the most recent wave of multimodal and generative AI developments, although it risks omitting foundational algorithmic work from earlier years. Publications were omitted if they: (1) were not connected to prosthodontics or dental applications; (2) did not apply AI-based methodology; (3) were editorials, comments, or conference abstracts without adequate methodological information; (4) were not published in English; or (5) fell outside the predefined publication window without clear foundational relevance.

### 2.3. Information Sources

A comprehensive literature search was conducted in PubMed, Scopus, and Web of Science. Publications dated from 1 January 2023 to 4 March 2026 were included, with a limited number of pre-2023 foundational studies retained when necessary to contextualize key AI applications reported in the recent literature. This timeframe was selected to capture the most relevant technological advances in AI, particularly sophisticated deep learning models, generative artificial intelligence, and multimodal models in digital dentistry, while recognizing that foundational algorithms and earlier applications have been extensively documented in prior systematic reviews. Given the rapid evolution and obsolescence of AI technologies, emphasizing recent literature allowed the review to assess current state-of-the-art applications rather than outdated technical approaches.

Additional relevant studies were identified through backward citation tracking of the included articles and targeted supplementary searches for rapidly evolving topics that were incompletely captured by database indexing, particularly large language models and generative CAD architectures. Specifically, manual hand-searching targeted relevant peer-reviewed journals across dentistry and related dental sciences, including the Journal of Prosthetic Dentistry, Compendium of Continuing Education in Dentistry, Applied Sciences, Journal of Dental Sciences, and Digital Dentistry Journal, as well as relevant medical informatics repositories, to ensure the capture of the most recent advancements in generative AI architectures. All records identified through these supplementary methods were assessed using the same predefined eligibility criteria and two-stage screening procedure applied to database-derived records. Supplementary searching was discontinued when further citation tracking yielded no additional eligible studies within the predefined scope and publication timeframe. Seventeen of the 50 included studies were identified through supplementary citation tracking and targeted searches. Because these records were not retrieved through the primary database strategy, this approach is acknowledged as a potential source of selection bias.

### 2.4. Search Strategy

The search strategy was adapted to the syntax of each database. In PubMed, controlled vocabulary (MeSH) terms were used, whereas identical free-text terms were applied in the other databases. Searches were performed in the TITLE-ABS-KEY field in Scopus and in the TS field in Web of Science. Search terms were combined using Boolean operators (AND, OR). The complete search strategy, including all keywords, filters, and restrictions for each database, is presented in Table 1.

### 2.5. Study Selection Procedure

To minimize selection bias, all identified records were imported into Zotero (version 9.0.6; Corporation for Digital Scholarship, Vienna, VA, USA) for automated de-duplication. Screening was performed independently by two reviewers (D.A.B. and C.E.I.) in two stages: title and abstract screening to identify potentially relevant records, followed by full-text assessment against the predefined eligibility criteria. Disagreements regarding study inclusion were resolved by consensus or by consultation with a third, senior investigator (A.J.).

### 2.6. Data Collection Process

**Figure 1:**
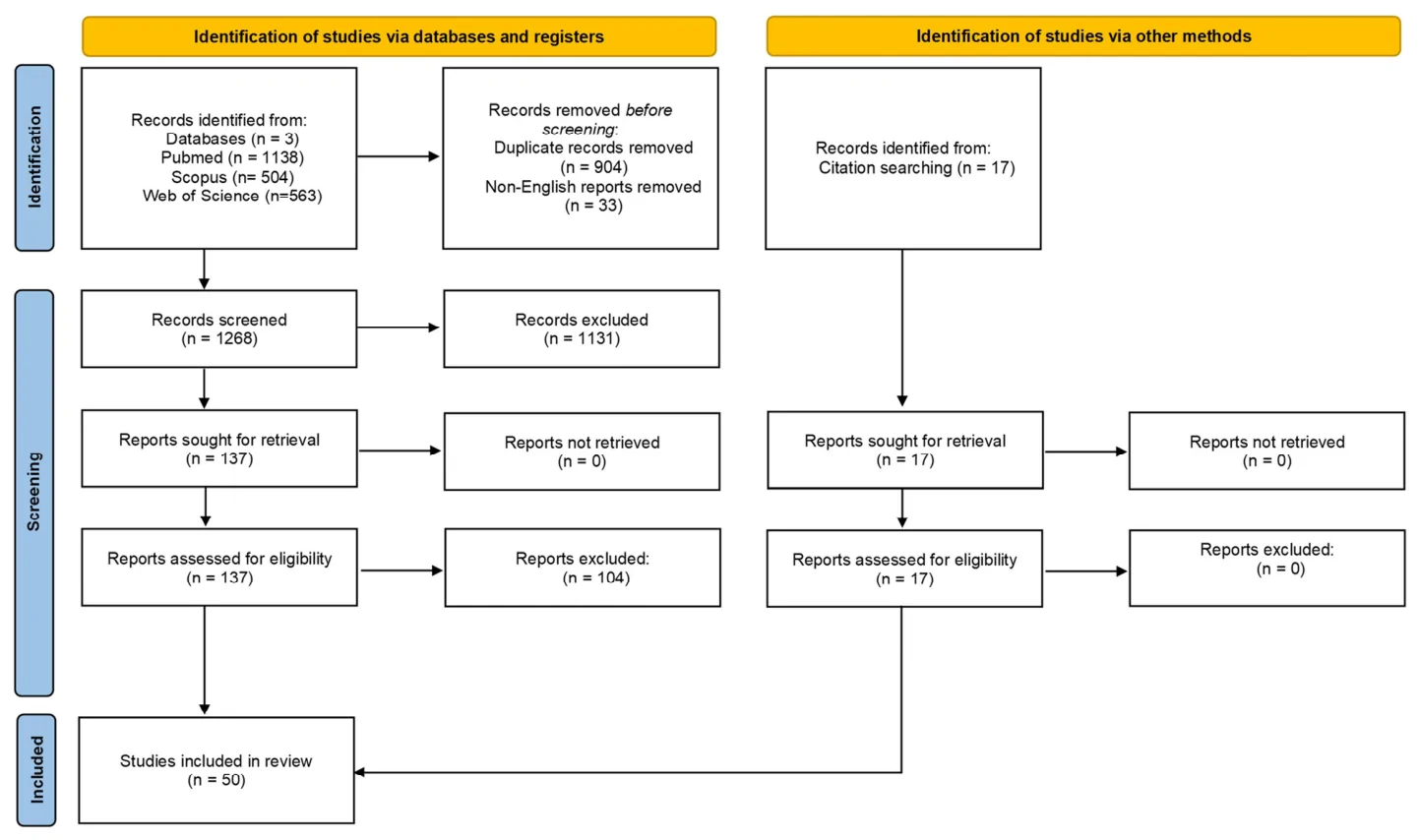
PRISMA Flow Diagram. Based on the PRISMA 2020 flow diagram template by Page et al. [13], licensed under the Creative Commons Attribution 4.0 International (CC BY 4.0) licence.

The literature search identified 2205 records from the selected databases. After duplicates were removed and language constraints applied, 1268 titles and abstracts were assessed, 1131 were discarded, and 137 full-text articles were evaluated for eligibility. Thirty-three studies identified through database searches met the inclusion criteria, and 17 additional studies were found using citation tracking and targeted manual web searches, yielding a final included sample of 50 studies. The PRISMA flow diagram illustrates the selection process (Figure 1).

### 2.7. Data Items

Data extraction was performed independently by two reviewers (D.A.B. and C.E.I.) using a standardized charting form. Any discrepancies during the data extraction phase were resolved through discussion and consensus, or by consulting the senior investigator (A.J.). The review sought to identify and delineate the primary domains of AI application in prosthodontics. Relevant information was succinctly summarized, encompassing the application types (e.g., digital workflows, CAD/CAM systems, intraoral scanning, implant dentistry, and esthetic analysis) and the computational role of AI in each domain.

### 2.8. Risk of Bias Assessment

According to PRISMA-ScR guidelines, a formal risk of bias assessment is not required for scoping reviews, as the main aim is to delineate existing literature rather than to evaluate it critically. As a result, no standardized quality assessment instruments (e.g., JBI critical appraisal checklists) were utilized. Nonetheless, while this study presents particular performance measures (e.g., accuracy and precision percentages) derived from research of differing methodological rigor, these statistics should be interpreted cautiously. The possible influence of this unexamined methodological bias on the claimed algorithmic efficacies is comprehensively discussed in Section 4.

### 2.9. Synthesis Methods

A qualitative narrative synthesis was performed. Studies were categorized by clinical application domain, including diagnosis and treatment planning, digital smile design, intraoral scanning and digital impressions, margin line detection and generation, CAD/CAM crown design and fabrication, color and shade identification, robotics, implant prosthodontics, and emerging educational applications. Because study designs and outcome measures varied substantially, quantitative synthesis was not performed.

To improve the reproducibility of the evidence maturity classification, each application domain was assigned to a maturity stage using predefined criteria based on the highest level of validation supported by the overall body of evidence. The categorization of each AI application into these specific maturity stages was determined through a consensus-based evaluation among the reviewers. Classification was performed independently by two reviewers, with disagreements resolved through discussion and consensus or consultation with the senior investigator. To ensure the reproducibility of our classifications, the following predefined criteria were applied: (1) Experimental: computational or laboratory proof-of-concept studies demonstrating feasibility without formal performance validation; (2) Technical Validation: formal performance evaluation using retrospective datasets or controlled in vitro models without prospective clinical validation; (3) Clinical Validation: existence of prospective, in vivo human trials assessing real-world performance; (4) Early Clinical Implementation: integration into real-world clinical workflows, including commercially available software platforms, but with limited evidence of clinical effectiveness, lacking widespread adoption; and (5) Routine Practice: standardized and regulated clinical integration supported by robust multicenter validation, demonstrated clinical effectiveness, and widespread adoption.

To strictly mitigate the risk of double counting primary data and the overrepresentation of specific findings, the systematic, scoping, and narrative reviews included in our analysis were utilized exclusively to map overarching clinical trends, systemic limitations, and implementation barriers. Quantitative data regarding algorithmic accuracy and operational efficiency were extracted from original research studies; to avoid methodological self-contradiction, any specific metrics initially cited from review articles were cross-referenced to ensure their validity, or generalized where the primary source was unavailable. It must be explicitly acknowledged that the high proportion of secondary reviews (24/50 studies) limits the overall strength of the underlying evidence base and highlights the scarcity of robust, original prospective clinical studies in this domain.

## 3. Results

### 3.1. Evidence Maturity Framework

**Figure 2:**
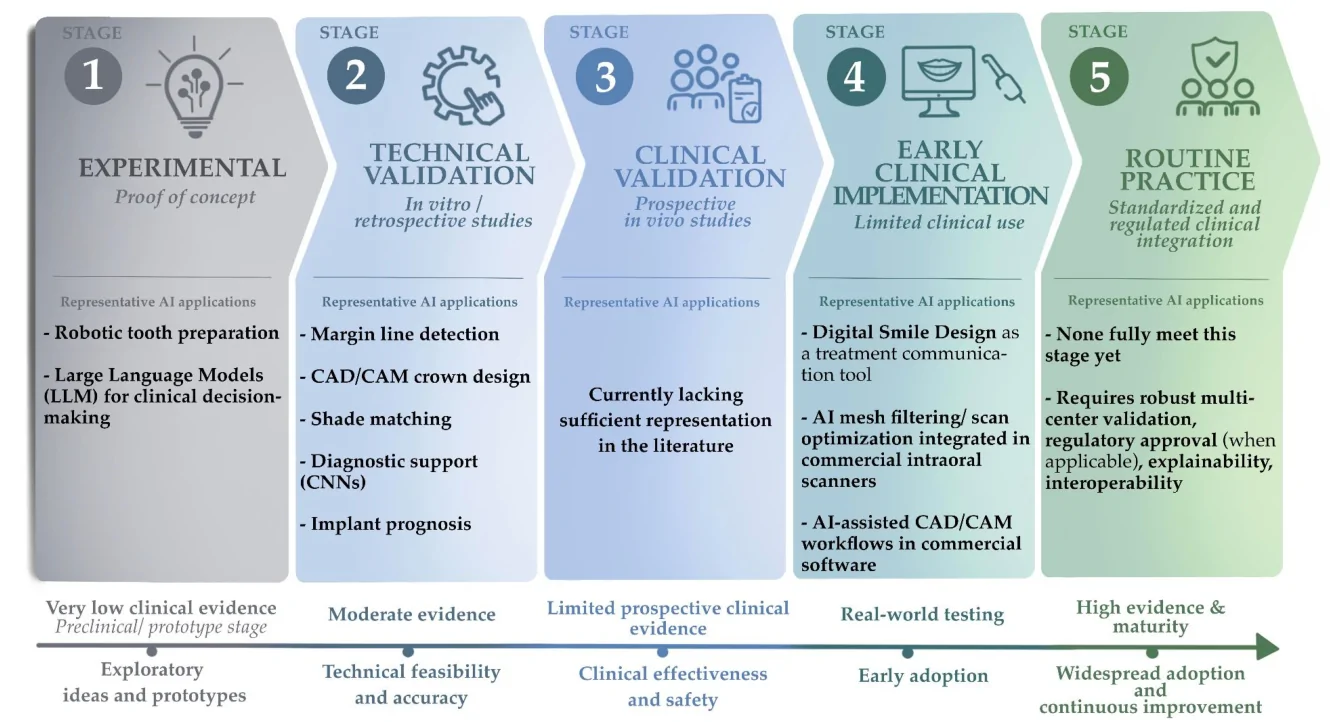
AI Maturity Roadmap in Prosthodontics. This roadmap reflects the maturity of clinical evidence and implementation in Prosthodontics. The colors distinguish the five maturity stages, while the arrow indicates progression toward increasing clinical evidence and maturity. Commercial availability alone does not imply routine practice.

To critically synthesize the literature rather than merely list studies, an Evidence Maturity Framework was applied to categorize the clinical readiness of AI applications identified in this review. Technologies were mapped across five distinct stages: (1) Experimental: Computational proof-of-concept without clinical validation; (2) Technical Validation: Retrospective or in vitro testing utilizing historical datasets; (3) Clinical Validation: Prospective in vivo testing assessing real-world performance; (4) Early Clinical Implementation: Early integration into clinical workflows or commercially deployed systems with early limited clinical use, lacking widespread adoption; (5) Routine Practice: Standardized and regulated clinical integration with widespread adoption. A graphical representation of this AI maturity roadmap across the various prosthodontic applications is summarized in Figure 2.

### 3.2. Study Selection

The selection process yielded a final sample of 50 studies for synthesis. To avoid repetition, the detailed screening sequence is reported in Section 2.6 and summarized in Figure 1.

### 3.3. Characteristics of Studies and Their Results

The research exhibited considerable methodological heterogeneity. Specifically, the 50 included records consist of 26 primary studies—comprising 8 clinical/in vivo investigations, 8 in vitro laboratory studies, and 10 computational algorithm validation studies—and 24 secondary literature records (systematic, scoping, and narrative reviews). Table 2 presents the characteristics of the included studies, including design, utilized AI architectures (such as CNNs, GANs, LLMs, etc.), clinical applications, and significant findings; review articles were included for contextual mapping, whereas quantitative performance metrics were interpreted from primary studies only. Table 3 presents a systematic overview of the primary application domains of AI in prosthodontics. The scoping synthesis of the literature was categorically organized into the following prosthodontic domains:

#### 3.3.1. Artificial Intelligence in Prosthodontic Diagnosis and Treatment Planning

##### Applications and Model Performance

The application of AI in prosthodontic diagnosis primarily encompasses two computational frameworks: machine learning algorithms for structured clinical data processing and deep learning algorithms, namely convolutional neural networks (CNNs), for radiographic analysis. Machine learning models are extensively employed for predictive analytics and decision-making support. The analysis of structured data, including demographics, clinical aspects, and radiographic information in tabular format, enables algorithms to predict the prognosis of periodontal disease, the risk of caries, and the long-term consequences of prosthodontic treatment [7].

Deep learning models have demonstrated their superiority in the automatic interpretation of radiographs across multiple applications. CNN architectures can deliver diagnostic predictions with remarkable accuracy, identifying cavities, periapical diseases, and alveolar bone loss with precision rates above those of seasoned clinicians [10,15]. A foundational example of this high technical efficacy is the CNN model examined by Görürgöz et al. (a work retained despite falling outside the 2023–2026 window due to its foundational relevance), which showed a sensitivity of 0.9867 and a precision of 0.7812 in automated tooth detection and numbering based on retrospective evaluations [14]. Building upon such foundational 2D detection, contemporary AI approaches now offer advanced 3D segmentation in CBCT and panoramic radiographs to independently identify healthy tooth structure, dental restorations, implants, and root canal fillings [11]. Furthermore, algorithms using deep learning with 3D intraoral scanners are also successfully used for dental arch categorization based on edentulism levels with good diagnostic reliability [5].

##### Critical Analysis and Limitations

While acknowledging the elevated accuracy of diagnostic models, a thorough examination of the included studies uncovers significant methodological issues. The majority of the diagnostic AI models were developed using limited and uniform datasets sourced from a single demographic group, resulting in concerns regarding algorithmic bias and inadequate generalizability to diverse populations [15]. These findings were primarily derived from controlled datasets and should not be interpreted as evidence of equivalent performance in routine clinical practice.

Moreover, there is an absence of prospective clinical trials that would juxtapose the efficacy of AI algorithms with the current gold standard (clinician evaluation) in clinical practice [8,15]. The opaque nature of deep learning networks poses a further obstacle to adoption, as the absence of explainability complicates the assessment of their judgments significantly [8].

##### Synthesis and Evidence Maturity

Current consensus confirms that CNN-based systems demonstrate consistently promising performance for well-defined image-based tasks. However, controversies persist regarding their real-world reliability, as methodological consistency is severely reduced by differences in datasets, imaging modalities, reference standards, and validation strategies. The implicit assumption that high algorithmic accuracy equates to clinical usefulness is flawed; panoramic-based bone models often disagree with clinical probing standards in real-world scenarios. It is important to note that the high diagnostic performance reported in this domain derives predominantly from retrospective or computational validation using controlled datasets rather than prospective clinical trials. Consequently, these performance metrics should be interpreted as indicators of technical efficacy rather than established real-world clinical effectiveness. The evidence therefore supports classification primarily at the technical validation stage. Robust prospective quality indicators, including patient-centered outcomes and comparisons with clinician performance under routine clinical conditions, remain limited. The principal unresolved gap is whether high diagnostic performance is maintained across heterogeneous patient populations and routine clinical conditions. Future research should prioritize prospective multicenter studies, external validation, standardized reference standards, and direct assessment of whether improved algorithmic performance translates into clinically meaningful treatment decisions.

#### 3.3.2. Digital Smile Design and Esthetic Treatment Planning

##### Current Applications and Workflow Integration

The integration of AI technology into Digital Smile Design (DSD) facilitates esthetic planning through automation, as opposed to manual assessments reliant on subjectivity. Utilizing computer vision technology, AI algorithms can automatically detect facial landmarks, analyze symmetry, and compute the appropriate proportions of teeth [3,17,18]. A 3D esthetic analysis workflow demonstrated high consistency and greater precision than conventional 2D analysis for tooth-related esthetic parameters, establishing a foundation for future AI-based automated esthetic assessment [19]. A significant accomplishment in the field is AI’s capability to integrate multimodal databases and overlay 2D extraoral pictures with 3D intraoral scans and CBCT images [7,16]. This combination is particularly relevant for difficult full arch implant rehabilitation due to the absence of natural references. Building on these multimodal capabilities, AI-driven workflows can now automatically generate facially driven virtual dental setups, reducing reliance on analog records and streamlining digital prototype development [20]. This diagnostic shift is further supported by evidence that 3D AI analysis of esthetics may overcome limitations associated with conventional 2D techniques [22], and that the recent implementation of 4D technology enables AI algorithms to identify and assess the dynamic movements of a smile [23].

##### Critical Analysis and Limitations

Despite the considerable decrease in time allocated to smile design and the high acceptance rate of generated designs in symmetric cases, clinicians favor manually crafted designs in asymmetrical situations, indicating that current AI templates fall short of the artistic proficiency of seasoned prosthodontists [21]. The methodological precision of AI-based DSD is significantly constrained by the stringent standardization of the image acquisition process required for this technology. Minor alterations in the patient’s head position or illumination circumstances may result in misalignments when integrating 2D and 3D information [19,20]. Furthermore, generic templates fail to account for cultural variations in the perception of attractive faces, necessitating ongoing oversight and supervision by physicians, along with stringent ethical norms regarding the preservation of biometric data [21,24]. Consistent with the narrative quality appraisal applied throughout this review, it should be noted that the reported acceptance rates and consistency gains for AI-assisted DSD originate largely from small, controlled clinical series or computational simulations rather than large prospective clinical cohorts, and should be interpreted accordingly.

##### Synthesis and Evidence Maturity

Current evidence for AI-assisted digital smile design is promising but clinically limited, with studies supporting improved workflow efficiency, visualization, and patient communication rather than clear superiority over clinician-guided planning. Methodological consistency remains limited because available investigations use different platforms, esthetic criteria, and outcome measures. Much of the available evidence is derived from small clinical samples, controlled evaluations, technique reports, or secondary literature; therefore, robust quality indicators for long-term clinical effectiveness remain insufficient. Accordingly, AI-assisted DSD is best positioned at early clinical implementation as an adjunctive visualization and treatment-planning system rather than an autonomous esthetic planning tool. Important knowledge gaps include performance in facial asymmetry, diverse facial phenotypes, and the relationship between AI-generated designs and long-term patient-reported outcomes. Future studies should use standardized esthetic outcomes, larger prospective cohorts, and diverse populations and should compare AI-assisted and clinician-guided workflows directly.

#### 3.3.3. Intraoral Scanning and Digital Impressions

##### Current Applications and Workflow Integration

AI algorithms integrated into intraoral scanning systems can facilitate the optimization of the meshing process. Specifically, AI-driven computer vision technologies autonomously eliminate moving soft tissues (such as the tongue or cheeks) and rectify minor imperfections throughout the meshing process [25]. Consequently, in vitro evidence suggests that AI assistance during complete-arch intraoral scanning can improve scan precision and reduce arch distortion compared with scanning performed without AI assistance [26]. Beyond intraoral data optimization, AI technology is extensively utilized in facial scanners to facilitate the automatic alignment and superimposition of extraoral facial structures with intraoral impressions [25,27].

##### Critical Analysis and Limitations

The implementation of AI-driven autonomous mesh repair functions introduces certain dangers in clinical practice. Automated data filling must be executed with utmost precision in key areas, such as the edges of prepared teeth, as minor algorithmic errors can affect the marginal fit of the final restoration [25]. Furthermore, the efficacy of AI algorithms may differ among various proprietary IOS devices; therefore, the accuracy outcomes should not be generalized [25,26].

##### Synthesis and Evidence Maturity

Evidence for AI-assisted intraoral scanning is moderate but predominantly technical, demonstrating improvements in mesh processing, artifact removal, and scanning precision under controlled conditions. Methodological consistency is restricted by scanner-specific algorithms, acquisition protocols, and experimental conditions, limiting generalizability between systems. Although AI-based processing is already incorporated into commercial scanning workflows, independent prospective evidence demonstrating improved patient-level or restoration-level clinical outcomes remains limited. Therefore, the available quality indicators primarily reflect technical scanning performance rather than established clinical effectiveness, placing this domain at early clinical implementation. Key gaps concern performance around subgingival margins, the consequences of automated mesh modification, and scanner-specific variance. Future research should prioritize prospective head-to-head evaluations of AI-enabled and conventional scanning, cross-platform validation, and clinically relevant outcomes such as restoration fit, rescanning requirements, chairside time, and treatment success.

#### 3.3.4. Automated Margin Line Detection and Generation

##### Current Applications and Model Performance

Extracting the margin line of a tooth from a 3D digital model is highly operator-dependent; therefore, a growing body of recent studies has applied 3D deep learning algorithms and mesh-based neural networks to automate this process. By training on large datasets of digital tooth models, these collective frameworks have achieved results approaching expert human performance [28,30]. Consistently across comparative analyses, deep learning models predicted margin lines with deviations of approximately 194–200 μm from expert-defined reference lines in computational validation studies, a range generally considered clinically acceptable [28,29].

##### Critical Analysis and Limitations

Automated margin detection remains in an early stage of clinical translation. Current models are less reliable when margin lines are poorly defined, when posterior anatomy is complex, or when scans contain artifacts [29]. Because many deep learning frameworks are trained on idealized tooth preparations, their performance may decline in suboptimal preparations or lower-quality scans [28,30]. Broader validation across diverse clinical scenarios is therefore required [31,32].

##### Synthesis and Evidence Maturity

AI-based margin line detection represents one of the more technically developed prosthodontic applications, with moderate evidence of reproducible performance under controlled conditions. Nevertheless, methodological heterogeneity remains substantial because studies differ in preparation geometry, scanning datasets, annotation protocols, algorithms, and definitions of ground truth. Most reported quality indicators derive from computational or in vitro validation rather than prospective clinical testing; consequently, high segmentation or detection performance should not be interpreted as equivalent performance in routine clinical conditions. This application therefore remains predominantly at the technical validation stage. The literature identifies a clear unresolved clinical problem: AI models trained on idealized, supragingival preparations routinely fail when tasked with extracting subgingival margins complicated by blood, saliva, or convex/concave boundary overlaps. Prospective clinical validation, standardized annotation protocols, external datasets, and direct comparisons with experienced technicians are therefore priorities for future research.

#### 3.3.5. AI-Assisted CAD/CAM Crown Design and Fabrication

##### Current Applications and Model Performance

The integration of AI, including Generative Adversarial Networks and 3D CNNs, into CAD/CAM systems supports the automated design of anatomically accurate dental crowns across several proprietary architectures. Alongside established platforms such as Dentbird (Imagoworks Inc., Seoul, Republic of Korea) and 3Shape (3Shape A/S, Copenhagen, Denmark), integrated clinical workflows, including Planmeca CAD/CAM systems (Planmeca Oy, Helsinki, Finland), are increasingly adopting AI-driven modules for margin detection and initial morphological proposals. These models analyze adjacent teeth, occlusal relationships, and available restorative space to generate crown designs [3,6,34]. Across multiple evaluations, there is strong consensus that AI-integrated design is substantially faster than conventional digital design, reducing design time by approximately 52% [33,35]. Beyond mere speed, generated crowns also demonstrate improved fit and precision; for example, GAN-based crowns showed a 35% lower internal fit discrepancy than conventionally designed crowns (55.4 vs. 85.6 µm RMS; *p* < 0.001) [34]. However, these findings have been demonstrated predominantly under controlled laboratory conditions, with limited prospective clinical validation.

##### Critical Analysis and Limitations

Furthermore, a narrative quality appraisal of the included literature reveals that impressive metrics—such as the 55.4 µm internal gap—derive largely from strictly controlled in vitro or computational proof-of-concept studies. Additional long-term in vivo clinical trials are necessary to assess whether these laboratory accuracies reliably translate to complex clinical settings [6,37].

##### Synthesis and Evidence Maturity

Among the reviewed applications, AI-assisted CAD/CAM crown design has relatively strong technical evidence, particularly for reducing design time and improving workflow reproducibility. Findings are reasonably consistent regarding efficiency, but less consistent for detailed morphology and functional reproduction, with current systems still showing limitations in reproducing complex occlusal anatomy and natural wear patterns. Because much of the supporting evidence remains in vitro, with only limited prospective clinical evaluation, this application is best classified predominantly at the technical validation stage, with emerging early clinical implementation through commercial platforms. Major knowledge gaps concern functional occlusion, individualized morphology, long-term restoration performance, and the extent to which reduced digital design time translates into meaningful chairside efficiency. An AI design that is 52% faster is irrelevant if the clinician must spend 20 min chairside adjusting the occlusion because the AI generated an unworn, idealized cusp. AI currently acts as an efficient draft-generator, not a final-prosthesis creator [36]. Future studies should prioritize prospective comparative trials evaluating clinical adjustment time, marginal and internal adaptation, occlusal performance, restoration longevity, and patient-centered outcomes.

#### 3.3.6. Artificial Intelligence for Color Detection and Matching

##### Current Applications and Model Performance

To overcome the subjectivity of visual shade guides, AI algorithms (including CNNs, Backpropagation Neural Networks, and Deep Neural Networks) are employed to extract spatial features from intraoral photographs and predict CIELAB color coordinates [11,39,40]. Among these approaches, advanced models utilizing a Grey Relational Analysis combined with a Deep Neural Network (GRA-DNN) achieved 93.2% accuracy in personalized tooth color recommendations, outperforming visual assessment (68.5%) and significantly reducing processing time [42]. Complementing these predictive models, other ML-based color correction strategies have proven highly effective at standardizing dental photographs across varying ambient lighting conditions to match VITA 3D-Master spaces [41]. Collectively, these specific AI algorithms have successfully lowered color differences (ΔE_00_) down to 1.0 in experimental in vitro settings, effectively matching strict clinical perceptibility criteria.

##### Critical Analysis and Limitations

Although AI applications serve as useful supportive tools, direct comparisons indicate that conventional clinical spectrophotometers surpass smartphone-based or solely photographic AI models in terms of absolute colorimetric accuracy [43]. The efficacy of AI shade matching is significantly influenced by external factors such as camera sensor quality, flash calibration, and tissue translucency, suggesting that it is not yet capable of operating as a totally autonomous diagnostic tool [38,39]. Consistent with the narrative quality appraisal applied throughout this review, headline performance metrics, including 93.2% accuracy and ΔE_00_ values down to 1.0, were obtained under controlled, standardized photographic or computational conditions rather than routine clinical settings, and their translation to variable real-world lighting and clinical environments remains limited.

##### Synthesis and Evidence Maturity

Evidence for AI-assisted shade selection is moderate and increasingly clinically relevant, with several approaches demonstrating promising agreement with objective color measurements. However, methodological consistency remains limited because studies use different image-acquisition conditions, AI architectures, shade systems, calibration procedures, and reference standards. Moreover, the available quality indicators are not uniformly comparable across studies, and conventional spectrophotometry remains superior in some direct comparisons. Although prospective clinical evidence is beginning to emerge, much of the supporting literature remains computational or in vitro; therefore, AI-based shade selection remains primarily at the technical validation stage. Remaining knowledge gaps include robustness to illumination, camera characteristics, tooth translucency, surface conditions, and population diversity. Performance is heavily compromised by ambient lighting and camera hardware variability. Future studies should employ standardized color protocols, spectrophotometric reference standards, larger prospective clinical cohorts, and external validation across different devices and clinical environments.

#### 3.3.7. Robotics and AI-Assisted Tooth Preparation

##### Current Applications and Model Performance

Robotic systems in prosthodontics, guided by AI spatial computing, aim to standardize tooth preparation. The most advanced systems utilize multi-axis robotic arms coupled with femtosecond laser photoablation. Guided by AI trajectory planning and digital models, these systems can autonomously remove carious tissue and prepare teeth for crowns or veneers with convergence angles and depths that closely match predefined CAD specifications, minimizing the risk of iatrogenic damage [44].

##### Critical Analysis and Limitations

Robotic tooth preparation is still confined to laboratory settings or carefully regulated experimental conditions due to significant clinical obstacles. These systems encounter difficulties in dynamically adjusting to abrupt patient movements, salivary contamination, and interference from soft tissues (tongue/cheek) [7,44]. Moreover, procedural efficiency remains limited; in vitro investigations indicate preparation times beyond 17 min per individual tooth [44]. The high price of equipment and the safety issues associated with lasers render robotic preparation far from being a normal clinical practice.

##### Synthesis and Evidence Maturity

The current evidence for AI-assisted robotics in prosthodontics is weak to moderate and predominantly experimental, with available studies demonstrating mechanical precision and technical feasibility rather than established clinical effectiveness. Methodological consistency is limited by heterogeneous robotic platforms, procedural tasks, experimental models, and performance criteria, while much of the evidence remains in vitro. Consequently, robotic tooth preparation and related applications should currently be classified at the experimental stage. Major gaps include patient safety, procedural efficiency, adaptation to anatomical variability and movement, comparative clinical performance, cost-effectiveness, and integration into existing workflows. Future research should progress from controlled laboratory validation toward prospective human studies evaluating accuracy, adverse events, treatment time, clinician intervention, and clinically meaningful outcomes. It must be explicitly stated that this domain currently lacks sufficient standardized clinical quality indicators; therefore, the reported performance metrics reflect computational benchmarking or laboratory conditions rather than validated clinical outcomes.

#### 3.3.8. Artificial Intelligence in Implant Prosthodontics

##### Current Applications and Model Performance

AI architectures applied to implant prosthodontics excel in radiographic segmentation and predictive analytics. Deep learning models applied to CBCT scans automate bone density assessments, map the mandibular canal, and simulate optimal implant placement trajectories [4,46]. Parallel to these volumetric analyses, in diagnostic imaging, AI algorithms demonstrate exceptional accuracy—often surpassing human experts—in identifying specific commercial implant brands from 2D periapical radiographs [46,47]. Building on both diagnostic and planning capabilities, ML predictive models have been developed to forecast osseointegration success and peri-implantitis risks, with prognostic accuracies ranging between 62.4% and 80.5% based on demographic and surgical variables [47,48].

##### Critical Analysis and Limitations

The wide variance in prognostic accuracy (62.4–80.5%) highlights the inconsistency and limited reliability of current predictive models [48]. AI implant planning tools remain strictly supplementary visual aids; they cannot autonomously dictate surgical decisions [46]. Primary studies reported high diagnostic accuracy for selected implant-related tasks, although most models were developed using retrospective datasets and require further external validation before widespread clinical implementation. Widespread clinical adoption is hindered by the need for massive, high-quality CBCT datasets for training, as well as strict regulatory barriers regarding automated surgical guidance systems [4].

##### Synthesis and Evidence Maturity

There is general consensus that AI applications in implant prosthodontics provide robust technical assistance for tasks including planning, classification, and biomechanical prediction. Conversely, significant controversies surround prognostic models, where clinical evidence remains comparatively limited and heavily reliant on biased retrospective datasets.

AI applications in implant prosthodontics demonstrate moderate technical evidence for tasks including prognosis, planning, classification, and biomechanical prediction, but clinical evidence remains comparatively limited. Methodological consistency is constrained by retrospective datasets, heterogeneous patient populations, differing outcome definitions, and limited external validation. Although high performance has been reported for selected implant-related tasks, the available quality indicators are derived predominantly from retrospective or computational analyses and therefore provide limited evidence of prospective clinical effectiveness. The range of predicted long-term implant survival (62.4–80.5%) further illustrates the limitations of retrospective datasets that do not consistently incorporate comprehensive patient comorbidity and clinical data. Most predictive applications therefore remain at the technical validation stage, despite increasing integration of AI-assisted planning into digital workflows. Important knowledge gaps concern prospective prediction of implant complications and survival, generalizability across populations and implant systems, and whether AI-assisted recommendations improve patient outcomes beyond conventional clinical assessment. Future research should prioritize longitudinal multicenter cohorts, standardized outcome definitions, external validation, and prospective comparisons between AI-assisted and conventional implant treatment planning.

#### 3.3.9. Emerging Applications: Large Language Models and Educational Support

##### Current Applications and Model Performance

Generative AI and Large Language Models (LLMs)—such as ChatGPT (GPT-3.5 and GPT-4; OpenAI, San Francisco, CA, USA), Gemini (Gemini Advanced; Google LLC, Mountain View, CA, USA), and Microsoft Copilot (GPT-4; Microsoft Corporation, Redmond, WA, USA)—are increasingly explored strictly as educational and communication aids rather than autonomous clinical decision-making tools [49,50]. When evaluated across these educational domains, a comparative analysis of chatbots answering prosthodontic questions showed that Microsoft Copilot (GPT-4) achieved the highest accuracy (73%), with the models performing best in implantology (75% accuracy) and poorest in removable partial prosthodontics (50.8%) [50].

##### Critical Analysis and Limitations

The clinical utility of conventional LLMs is severely compromised by “hallucinations”—the generation of highly confident but factually incorrect information or fabricated academic citations [52]. Studies explicitly warn that ChatGPT and similar models exhibit low reliability and repeatability in generating safe prosthodontic treatment advice [51]. Despite the potential of Retrieval-Augmented Generation (RAG) systems grounded in validated medical literature to reduce fabricated information, LLM-generated content still requires independent verification before it can be considered reliable for patient-facing clinical decision-making [52].

##### Synthesis and Evidence Maturity

The evidence supporting LLMs in prosthodontics is currently limited and methodologically inconsistent, with performance varying substantially according to model, question type, prompting strategy, and evaluation method. Existing quality indicators primarily consist of benchmark accuracy, expert assessment, and response-based evaluations rather than prospective clinical outcomes; consequently, there is insufficient evidence to establish their safety or effectiveness in patient-facing clinical decision-making. Although these systems show potential for education and communication, hallucinations, factual inaccuracies, and limited repeatability substantially restrict their clinical reliability. Accordingly, conventional LLMs remain at the experimental/educational stage and should not currently be regarded as autonomous clinical decision-support systems. Critical knowledge gaps include reproducibility, source traceability, performance on complex patient-specific scenarios, and the effectiveness of domain-grounded architectures. Future research should prioritize standardized clinical benchmarks, expert-validated datasets, Retrieval-Augmented Generation using authoritative medical sources, prospective clinician–AI studies, and rigorous assessment of safety, explainability, and hallucination rates.

## 4. Discussion

The findings of this scoping review indicate that AI has become an increasingly important component of the digital transition in prosthodontics, particularly in image analysis, prosthetic design, workflow automation, and educational support. Across the included primary studies, CNN-based models showed strong performance in diagnostic and segmentation tasks, while GAN-based and related generative architectures demonstrated value in crown morphology generation and CAD/CAM optimization [14,34]. AI demonstrated the greatest success for well-defined image-based tasks, whereas performance became progressively less consistent for esthetic judgment and complex clinical reasoning [14,21,52]. These results are in agreement with the general trends reported in the systematic and narrative reviews included in this research, which also identify image analysis and digital workflow optimization as the most advanced applications of AI in prosthodontics [3,9,10].

Although the overall findings support the growing role of AI in prosthodontics, negative and inconsistent findings—though less frequently reported—underscore the need for caution when considering near-term clinical translation. AI-assisted digital smile design improved treatment planning and patient communication, yet did not consistently outperform clinician-guided workflows in patients with facial asymmetry [21,22]. Likewise, while AI-based shade matching demonstrated promising accuracy, spectrophotometric methods remained superior in some comparative studies [43]. AI-assisted CAD/CAM systems consistently reduced design time, although current algorithms still struggle to reproduce complex occlusal anatomy and natural wear patterns [34,36]. The greatest variability was observed for large language models, which demonstrated marked differences in accuracy across models and prosthodontic domains, while persistent hallucinations further limited their clinical reliability [50,52]. These discrepancies likely reflect differences in study design, validation methods, datasets, and evidence maturity. Importantly, the relative scarcity of explicitly reported failures or unsuccessful clinical translations across the included literature is itself noteworthy and may reflect a broader publication bias toward favorable results.

A persistent gap remains between computational performance and clinical readiness. High accuracy reported in controlled in vitro or retrospective settings cannot be assumed to translate directly into routine care, where saliva, blood, patient movement, subgingival preparations, heterogeneous imaging conditions, and operator-dependent variables may affect model performance [25,28]. According to the evidence maturity framework applied in this review, most AI applications in prosthodontics remain at the technical validation stage rather than routine clinical practice. The studies included in this review repeatedly demonstrated promising performance of artificial intelligence (AI) systems; however, the strength of the current evidence remains limited by the predominance of retrospective and laboratory-based investigations rather than prospective clinical validation. The majority of existing evidence is based on datasets from a single institution or imaging system, with limited external or prospective validation. These methodological limitations increase the risk of overfitting and spectrum bias, potentially resulting in overly optimistic estimates of real-world performance. Thus, published performance indicators may not fully reflect the effectiveness of AI systems under routine clinical conditions. In this context, it should be emphasized that commercial availability of an AI-enabled feature is not equivalent to demonstrated clinical effectiveness; several tools reviewed in this manuscript are already integrated into commercially available CAD/CAM and intraoral-scanning software, despite the supporting evidence ranging from technical validation to early clinical implementation. This discrepancy highlights that commercial adoption may precede rigorous clinical validation.

A recurring fallacy in the reviewed literature is the implicit assumption that high algorithmic accuracy equates to clinical usefulness. Technical performance metrics (e.g., sensitivity, precision, ΔE) do not automatically translate into patient-centered clinical benefits. This clinical applicability gap highlights a critical discrepancy: while computational studies highlight benefits such as a 52% faster crown design time, these laboratory gains may not address the true bottlenecks clinicians face. For example, the primary challenges in digital workflows often revolve around intraoral scanning accuracy in deep subgingival margin areas, capturing dynamic occlusion, or the clinical chairside time required for manual occlusal adjustments—variables that current AI models do not fully resolve. In the context of LLMs, the reported metrics (e.g., 73% accuracy for Copilot) serve more as an educational cautionary tale than evidence of a clinical decision-making tool. LLMs are better regarded as educational and communication-support tools, as their propensity for hallucinations renders them unsuitable for autonomous clinical guidance.

The strongest evidence currently supports AI as a workflow-optimization and decision-support technology rather than as an autonomous clinical decision-maker. In CAD/CAM design, margin identification, shade selection, and radiographic interpretation, AI may reduce repetitive manual tasks, improve reproducibility, and limit some forms of observer variability [14,30,33,42]. However, human-AI collaboration is fundamental. Esthetic planning in asymmetrical faces, functional occlusal refinement, individualized morphology, and long-term biomechanical judgment continue to require the expertise of experienced prosthodontists, particularly when algorithmic outputs are based on generic templates or limited training data [21,33]. Current research suggests that AI has the potential to benefit prosthodontics, but it is not yet adequate to establish consistent clinical success across a range of practice settings and patient types. Human supervision remains essential, particularly in complex restorative cases requiring individualized clinical judgment.

Reproducibility also remains insufficiently addressed, as many reviewed studies provided insufficient detail regarding data preprocessing, model architecture, or validation strategies, while publicly accessible datasets and open-source code remained rare. There is considerable variety in imaging techniques, scanner technology, restorative materials, patient groups and outcome metrics, which affects the generalizability of recent findings. Differences in ground-truth definitions, such as margin lines annotated by different experts, further complicate direct comparisons between algorithms and make it difficult to determine whether differences in performance reflect superior models or more favorable testing conditions. Such limitations restrict independent verification of reported findings and contribute to the broader reproducibility concerns in contemporary AI research. Collectively, these constraints underscore the importance of prospective multicenter research, standardized reporting criteria, and external validation using different datasets before artificial intelligence systems can be regarded as reliable for widespread clinical adoption in prosthodontics.

Despite encouraging technological progress, several impediments continue to limit the clinical implementation of AI in prosthodontics. The potential underrepresentation of negative evidence may contribute to an overly optimistic perception of AI readiness, particularly when performance under unpredictable clinical conditions such as saliva, bleeding, patient movement, or atypical anatomies remains insufficiently characterized. Performance may decline during clinical deployment because models trained under standardized conditions encounter domain shifts related to different patient populations, imaging systems, acquisition protocols, and unpredictable intraoral conditions. Interoperability between proprietary CAD/CAM software, intraoral scanners, and AI platforms remains a major challenge and may restrict seamless integration into existing digital workflows. Economic sustainability is another concern, as software acquisition, licensing, hardware requirements, maintenance, and clinician training must be justified by measurable improvements in clinical efficiency or patient outcomes. Regulatory approval presents additional challenges because AI systems require evidence of safety, reliability, transparency, and consistent performance across different clinical settings. The European Union Artificial Intelligence Act and the GDPR requirements concerning patient data are expected to shape the future use of AI-powered prosthodontic software. Post-market surveillance, transparency, explainability and security are likely to be the focus of increased regulatory requirements.

Future studies should focus on prospective multicenter clinical trials, consistent reporting methodologies, publicly accessible benchmark datasets, external validation in different geographic populations, and head-to-head comparisons of commercial artificial intelligence (AI) systems. In the future, the development of “multimodal foundation models”—AI that can ingest text, radiographs, and 3D intraoral scans simultaneously—may enable a genuinely integrated clinical assistant. Greater attention should also be directed toward explainable AI. In complex prosthodontic cases, where esthetic and biomechanical considerations extend beyond numerical predictions, clinicians may find it difficult to validate AI-assisted decisions or identify algorithmic errors when model outputs are not interpretable. Generative AI also raises ethical issues around fabricated information, non-transparent content generation, authorship, and clinician overreliance. These tools should therefore be viewed as a complement to professional judgment, not as a replacement.

The conclusions of this review should be interpreted in light of the varying levels of evidence contained in the collected literature. Primary clinical and in vitro studies provided the foundation to assess the performance and possible clinical applicability of standard artificial intelligence (AI) systems, whereas narrative, systematic, and scoping reviews helped to provide context, synthesize broader research trends, and identify existing knowledge gaps. Distinguishing between these evidence types allows for a more balanced interpretation of the current state of AI in prosthodontics.

Finally, while scoping reviews do not strictly require a priori registration, the absence of a prospectively registered protocol remains a methodological limitation that we explicitly acknowledge.

## 5. General Limitations and Implementation Challenges

Despite substantial technical progress, several systemic barriers continue to limit the routine clinical implementation of AI in prosthodontics.

### 5.1. Data Quality, Algorithmic Bias, and Methodological Limitations

The included literature reveals recurrent methodological limitations that may overstate the apparent readiness of AI systems for clinical use. Many models were developed using relatively small, homogeneous, single-institution datasets, which increases the risk of overfitting and limits external validity. Consequently, these models suffer from “domain shift”—a phenomenon where algorithmic performance degrades significantly when applied to new populations, different ethnic demographics, or varying imaging hardware. Models trained on highly standardized intraoral scans, radiographs, or photographic datasets may not generalize reliably across diverse populations, imaging devices, acquisition protocols, and clinical conditions [14,15].

The current evidence base is also dominated by retrospective, computational, and in vitro studies. Consequently, the extent to which reported performance metrics reflect real-world clinical effectiveness remains uncertain. Publication bias may further amplify this limitation, because studies reporting high accuracy or favorable workflow gains are more visible than studies documenting algorithmic failure, reduced performance, or negative clinical consequences. Future research should therefore prioritize multicenter data-sharing networks, standardized reporting, and prospective validation under clinically realistic conditions.

### 5.2. The “Black Box” and Explainability

The lack of transparency in deep learning models (the “black box” phenomenon) presents a major barrier to professional trust. In prosthodontics, where treatment decisions have long-term biomechanical and esthetic consequences, clinicians must understand the rationale behind an AI recommendation. Therefore, high predictive performance alone may be insufficient to justify clinical implementation when the basis of an AI-generated recommendation cannot be adequately interpreted or independently verified by the clinician. The development of “Explainable AI” (XAI) is therefore a critical frontier for future research [8,45].

### 5.3. Ethical, Legal, and Educational Gaps

As AI assumes a greater role in prosthetic design, robotic preparation, and patient-facing informational support, ethical and legal questions become more pressing. Liability remains unclear when an algorithmic recommendation contributes to an incorrect design, inappropriate treatment plan, or clinical complication. Future integration requires continuous AI auditing, strict cybersecurity measures to protect biometric dental data, data ownership transparency, and clear medico-legal frameworks defining clinician accountability versus developer liability. In parallel, dental education must incorporate AI literacy so that clinicians can evaluate model limitations, recognize biased or unreliable outputs, and supervise AI-assisted workflows rather than accepting algorithmic recommendations uncritically [3,11,45].

### 5.4. Limitations of This Scoping Review

This review has several limitations. First, limiting the search to English-language publications may have excluded relevant international evidence. Second, because the field is evolving rapidly, technologies classified as emerging in 2023 may already have been superseded by newer systems by the time of publication. Furthermore, the integration of mixed study designs—such as combining primary research with systematic, scoping, and narrative reviews—represents a methodological limitation. The high heterogeneity of AI architectures and outcome measures across the 50 included studies precluded quantitative meta-analysis and limited the ability to generate pooled estimates of AI efficacy. Although strict data extraction stratification was applied to prevent the duplication of evidence, this approach may still introduce a potential risk of bias.

Additionally, relying on manual citation tracking to identify 34% of the included studies (*n* = 17) introduces a potential risk of selection bias. Although this approach was necessary to capture emerging technologies like LLMs and generative CAD tools, these records were not retrieved through automated, syntax-based database searches.

Finally, a formal study protocol was not pre-registered in a public repository (e.g., PROSPERO or OSF). Although protocol registration is not mandatory for scoping reviews, its absence represents a constraint on methodological transparency, albeit mitigated by adhering strictly to PRISMA-ScR guidelines.

### 5.5. Future Research Directions

Based on the reviewed literature, several priorities are needed to translate computational progress into clinically reliable implementation:

Prospective Clinical Validation: A critical shift is required from retrospective, in vitro validations to multicenter, prospective randomized controlled trials (RCTs) to assess AI efficacy in dynamic real-world clinical settings.

Open-Source Multimodal Datasets: To address algorithmic bias, global consortiums should prioritize the creation of open-access, ethnically diverse, and multi-institutional datasets that include intraoral scans, radiographs, and clinical pictures.

Explainable AI (XAI): Future deep learning frameworks in prosthodontics should prioritize explainability by providing clinicians with visual or textual justifications, such as attention maps, for diagnostic or design outputs to strengthen professional trust.

AI Literacy in Dental Education: Academic institutions should incorporate digital data management and AI algorithm appraisal into prosthodontic curricula so that future clinicians can critically evaluate AI-generated recommendations.

## 6. Conclusions

Artificial intelligence is increasingly influencing the field of prosthodontics. Contemporary computational models have demonstrated promising performance in diagnostic tasks, CAD/CAM workflows, and esthetic applications; however, evidence of workflow improvement is derived predominantly from laboratory, computational, and retrospective studies. Consequently, these reported benefits should not yet be interpreted as evidence of equivalent improvements under routine clinical conditions. The limited representation of unsuccessful or negative validation outcomes in the included literature restricts a balanced assessment of current AI performance and clinical readiness. Rigorous prospective in vivo research is required to establish whether these technological advantages observed under controlled conditions translate into meaningful improvements in clinical efficiency, treatment quality, and patient outcomes. Until such robust evidence is established, clinicians must maintain a highly cautious interpretation of AI clinical readiness.

Moreover, broad implementation of these technologies requires robust regulatory and ethical frameworks. Dentistry must establish clear protocols to protect patient safety and define practitioner responsibility when algorithm-driven workflows are used. Persistent explainability limitations remain an additional barrier, as clinicians may be unable to adequately interpret or independently verify AI-generated output, further warranting caution regarding the clinical readiness and implementation of current AI systems. Because current algorithms remain unable to provide fully autonomous, context-sensitive decision-making, continuous clinical supervision is essential.

These conclusions should be interpreted considering the study’s methodological constraints, including the lack of a priori protocol registration and the inclusion of mixed study designs, which underline the need for future standardized, prospective validation.

Ultimately, computational models should augment rather than replace human expertise by enabling more individualized and efficient care. They cannot substitute for the clinical judgment, tactile skill, and esthetic sensibility of an experienced prosthodontist. The future of the field will depend on effective human–machine collaboration to improve patient outcomes. Future research should move beyond isolated algorithmic optimization and prioritize multicenter prospective validation, standardized reporting frameworks, implementation studies conducted under real-world clinical conditions, and the establishment of robust regulatory governance.

## Figures and Tables

**Table 1:** Search strategies applied across selected databases.

PubMed
(“Artificial Intelligence”[Mesh] OR “Artificial Intelligence”[tiab] OR “AI”[tiab] OR “Machine Learning”[Mesh] OR “Machine Learning”[tiab] OR “Deep Learning”[Mesh] OR “Deep Learning”[tiab] OR “Neural Networks, Computer”[Mesh] OR “Neural Network*”[tiab] OR “Computational Intelligence”[tiab] OR “Automation”[Mesh] OR “automation”[tiab] OR “automated”[tiab] OR “Robotics”[Mesh] OR “robotic*”[tiab]) AND (“Prosthodontics”[Mesh] OR “prosthodontic*”[tiab] OR “Digital Smile Design”[tiab] OR “intraoral scanning”[tiab] OR “intraoral scanner*”[tiab] OR “digital impression*”[tiab] OR “CAD-CAM”[Mesh] OR “CAD/CAM”[tiab] OR “computer-aided design”[tiab] OR “computer-aided manufacturing”[tiab] OR “crown design”[tiab] OR “dental crown*”[Mesh] OR “dental crown*”[tiab] OR “color detection”[tiab] OR “shade selection”[tiab] OR “implant dentistry”[tiab] OR “dental implants”[Mesh] OR “dental implant*”[tiab])
Scopus
TITLE-ABS-KEY((“artificial intelligence” OR “machine learning” OR “deep learning” OR “neural network*” OR “computational intelligence” OR AI) AND (prosthodontic* OR “digital smile design” OR “intraoral scanning” OR “intraoral scanner*” OR “digital impression*” OR “CAD/CAM” OR “computer-aided design” OR “computer-aided manufacturing” OR “dental crown*” OR “shade selection” OR “implant dentistry” OR “dental implant*”)) AND (LIMIT-TO (SUBJAREA, “DENT”)) AND (LIMIT-TO (PUBYEAR, 2023) OR LIMIT-TO (PUBYEAR, 2024) OR LIMIT-TO (PUBYEAR, 2025) OR LIMIT-TO (PUBYEAR, 2026)) Results were limited to Scopus category Dentistry.
Web of Science
TS=( (“artificial intelligence” OR AI OR “machine learning” OR “deep learning” OR “neural network*” OR “computational intelligence” OR automation OR automated OR robotic*) ) AND TS=( (prosthodontic* OR “digital smile design” OR “intraoral scanning” OR “intraoral scanner*” OR “digital impression*” OR “CAD/CAM” OR “computer-aided design” OR “computer-aided manufacturing” OR “crown design” OR “dental crown*” OR “color detection” OR “shade selection” OR “implant dentistry” OR “dental implant*”) ) Results were limited to the Web of Science category Dentistry, Oral Surgery & Medicine.

* denotes a wildcard operator used to retrieve multiple word variants sharing the same root.

**Table 2:** Characteristics of included studies.

No.	Author (Year)	Type of Study	Validation Method	AI Technique/ Model	Dataset Size	Application Area	Key Findings
1	Schwendicke et al. (2025) [7]	Review	N/A	ML, DL, computer vision, generative AI	N/A	General prosthodontics	Identifies rapid growth in AI-automated crown design and digital manufacturing.
2	Blatz (2025) [3]	Review	N/A	ML, DL	N/A	Clinical dentistry	Describes AI as a key driver of precision dentistry through improved diagnosis, treatment planning, and CAD/CAM integration.
3	Inchingolo et al. (2025) [10]	Systematic review	N/A	ML, DL, CNN	15 clinical studies analyzed	Dental diagnostics	Confirms diagnostic efficacy for caries and bone loss; notes small training set limits.
4	Samaranayake et al. (2025) [1]	Narrative review	N/A	CNN, ANN, GAN LLM, VLM, MMs	N/A	General dentistry	Highlights the potential of LLMs and multimodal vision transformers for diagnostics.
5	Görürgöz et al. (2022) [14]	Retrospective Accuracy Study	Computational/In vitro	Faster R-CNN (GoogLeNet Inception v3 backbone)	1686 periapical radiographs	Dental Charting	Successfully automated jaw classification and tooth numbering.
6	Vashisht et al. (2024) [2]	Scoping review	N/A	ML, DL, ANN, CNN	87 articles analyzed	General dentistry	Identifies a positive correlation between dataset size and diagnostic precision.
7	Lee et al. (2025) [4]	Narrative review	N/A	CNNs, GANs, Transformers, Diffusion Models	120 articles reviewed	General dentistry	AI improves dental care and management, though bias and privacy challenges remain.
8	Aljulayfi et al. (2024) [5]	Systematic review	N/A	AI, ML, DL, ANNs, CNNs, R-CNN, GANs	30 studies analyzed	Prosthodontics	AI-assisted fixed prosthetic planning improves clinical accuracy vs. visual methods.
9	Dawood et al. (2026) [6]	Scoping review	Software Audit	DL, ANNs, CNNs, GANs, INN	46 studies & 16 CAD/CAM software platforms	Digital Prosthetic design/ CAD-CAM	Automated CAD packages improve morphological accuracy, design time, and reproducibility.
10	Rokhshad et al. (2026) [8]	Systematic review	N/A	CNNs, GANs, ANNs	31 studies analyzed	Fixed & Removable Prosthodontics	GANs show favorable performance in generating realistic restorations but lack multicenter validation.
11	Al Hendi et al. (2024) [9]	Narrative Review	N/A	ML, CNNs, ANNs	N/A	Prosthodontics	Synthesizes AI applications in margin line detection, shade picking, and implant stress analysis.
12	Najeeb and Islam (2025) [11]	Narrative Review	N/A	CNNs, SVMs, NNs	63 articles analyzed	Restorative dentistry	Reviews DL accuracy in diagnostics; highlights data privacy and bias as barriers.
13	Araidy et al. (2025) [15]	Systematic review	N/A	CNNs, ANNs, Trans-VNet	23 studies analyzed	General dentistry	Reports that 79% of current AI studies are retrospective, needing real-world validation.
14	Lv et al. (2025) [16]	Dataset Validation Study	Computational/Finetuning	Qwen-VL (3B & 7B models), GPT-4o	8775 checkups (50,000 photos, 8056 radiographs) from 4800 patients	Multimodal oro-dental data/ diagnostics	Introduces the landmark multimodal COde dataset that improves AI-assisted diagnostic report generation.
15	Saini et al. (2025) [17]	Systematic review and meta-analysis	N/A	ML, DL, AI-based DSD	7 studies (including a survey of 628 subjects)	Esthetic Dentistry/Digital smile design	Concludes that AI-based DSD improves smile symmetry and patient satisfaction.
16	Dhopte and Bagde (2023) [18]	Narrative Review	N/A	ML, DL, CV, robotics, VR/AR	N/A	Digital dentistry	Highlights the broad applicability of AI for diagnosis, treatment planning, robotics, and virtual dentistry.
17	Cheung et al. (2024) [19]	Controlled In Vitro Study	Computational/In vitro	AI-assisted 3D workflow, CV, Mathematical Modeling/Coordinate Mapping	15 healthy patients (facial scans + intraoral models)	Esthetic analysis/DSD	Demonstrates that AI-assisted 3D virtual-patient creation provides greater consistency than conventional 2D approaches.
18	Lawand et al. (2026) [20]	Clinical Technique Report	Retrospective clinical	AI Smile Design Platform (Smilecloud)	1 fully edentulous patient	Smile design/implant-supported rehabilitation	Demonstrates the feasibility of AI-assisted digital workflows for complete-arch implant rehabilitation.
19	Ceylan et al. (2023) [21]	Cross-Sectional Survey	Controlled clinical survey	Microsoft Face API (68 landmark detection engine)	4 clinical cases evaluated by 628 survey participants	Esthetic Analysis/DSD	AI-generated smiles are well accepted for symmetric faces; manual preferred for asymmetric.
20	Baaj (2025) [22]	Scoping review	N/A	DL, CV, CNNs	4 core articles analyzed	Esthetic Dentistry/DSD	3D AI smile mapping avoids 2D distortions; esthetic perceptions are not significantly different from manual designs.
21	Georg (2023) [23]	Clinical Technique Report	Retrospective clinical	Facial Analysis, DSD	1 young female patient case	Esthetic analysis/DSD	Outlines digitally driven crown-lengthening and vertical dimension shifts.
22	Rokhshad et al. (2024) [24]	e-Delphi consensus Study	Controlled clinical consensus	AI image processing, simulation AI, predictive AI	28 international ITU/WHO experts	Bioethics/AI Governance	Establishes bioethical guidelines; AI must not be an over-treatment marketing tool.
23	Alghauli et al. (2025) [25]	Comprehensive Narrative Review	N/A	DL, CV, predictive AI	N/A	Digital acquisition, CAD/CAM	AI supports faster and more automated digital acquisition through scan cleaning, alignment, and CAD/CAM integration; performance remains dependent on clinical conditions.
24	Róth et al. (2025) [26]	Controlled In Vitro Study	In vitro	3Shape Trios 5 AI Mesh-Filtering Engine	3 articulated Type IV gypsum arches (120 total scans)	Intraoral scanning/digital impressions	Enabling AI mesh-filters optimizes global scanning precision and reduces distortion.
25	Revilla-León et al. (2024) [27]	Overview/review	N/A	DL, CV, segmentation/alignment AI	N/A	Digital data acquisition and implant planning	Highlights AI-assisted automation of scan processing, alignment, and implant planning.
26	Alsheghri et al. (2026) [28]	Algorithm Development Study	Computational/In vitro	Modified MeshSegNet (18 geometric/curvature channels), ensemble learning	54 oral patient die models (incisors)	Margin line generation	AI successfully extracted incisor margin lines; performance depends on prep quality.
27	Alsheghri et al. (2025) [29]	Algorithm Development Study	Computational/In vitro	iMeshSegNet Architecture (with Adjacency Repair), ensemble learning	1113 digital surface scans of prepped teeth	Margin line extraction	AI successfully extracted margin lines; novel AI post-processing strategy outperformed standard graph-cut methods.
28	Alsheghri et al. (2024) [30]	Experimental study	Computational/In vitro	AdaPointTr Framework (with Local Density + PCA), ensemble learning, UQ	1047 intraoral scans (913 train, 134 test)	Margin line generation	Implements a point-cloud transformer to quantify AI prediction uncertainty.
29	Choi et al. (2024) [31]	Software Accuracy Comparison	Computational/In vitro	Dentbird Solution (CenterNet + UNext hybrid system)	182 jaw scans (Group DS cast scans, Group IS intraoral)	Margin line detection	Establishes clinical acceptance thresholds for AI-based margin detection, particularly in challenging subgingival regions.
30	Sawangsri et al. (2025) [32]	In vitro comparative study	In vitro	AI-based CAD, 3Shape Automate vs. Dentbird Crown 4.1.1	100 digital scans of natural prepped abutments	Margin line detection and contour design	AI-based CAD achieved acceptability scores comparable to human technicians.
31	Du et al. (2026) [33]	Fully Automated System Validation	Computational & Prospective Clinical	CrownSurfaceFormer (CSF) (with DEM + D-FCN)	110 patient intraoral scans	Occlusal surface design	AI successfully maps maxillary parameters to design mandibular molars; struggles with deep fissures.
32	Cho et al. (2023) [34]	In Vitro Comparative Evaluation	In vitro	Dentbird Crown (StyleGAN + Pixel2Style2Pixel encoder)	30 datasets of partial arch clinical scans	CAD/CAM crown design	AI provides major design work time advantages and superior internal fit adaptations.
33	Nagata et al. (2025) [35]	In Vitro Evaluation Study	In vitro	AI-based CAD, Dentbird AI CAD Engine	20 milled resin crowns designed by 5 technicians	Crown design	AI-CAD produces more consistent crown designs, highly uniform occlusal geometries and significantly faster design times.
34	Wang et al. (2025) [36]	In Vitro Morphological Analysis	In vitro	3Shape Automate	12 digital prepped casts (mandibular premolars)	Crown morphology	AI models structural diameters accurately but fails to replicate real cusp wear facets.
35	Liu et al. (2025) [37]	Mixed In Vitro & Prospective Human Trial	In vitro & Prospective clinical	Statistical-model AI for crown design	20 cast restorations + 6 clinical crowns placed in 3 patients	Zirconia crown design	AI-designed clinical restorations display favorable anatomical realism and marginal adaptation.
36	Binhuraib et al. (2024) [38]	Narrative Review	N/A	ML, DL	N/A	Shade matching	AI networks detect hidden color distribution areas.
37	Shetty et al. (2024) [39]	Systematic review	N/A	Fuzzy logic, BPNN, CNNs, ANNs, SVM, YOLO, RF/XGBoost	15 core studies analyzed	Shade matching	Synthesizes neural architecture accuracy in shade prediction; decision-tree regression achieves the highest shade prediction.
38	Bekdaş et al. (2025) [40]	In Vivo Model Development	Computational/In vivo validation	MobileNetV2, ResNet18, DenseNet121, EfficientNetB0	1031 tooth images from 102 participants	Color detection/shade selection	AI achieved high accuracy in mapping CIELAB parameters from uncalibrated extraoral photos against spectrophotometric baselines.
39	Li et al. (2025) [41]	In vitro experimental study	Computational/In vitro	Extreme Learning Machine (ELM), PR, BPNN	ColorChecker card and VITA 3D-Master guide photographs	Shade recommendation/color standardization	Validates a 2-tier AI color correction strategy falling below the clinical acceptability threshold.
40	Ruan et al. (2026) [42]	Experimental validation study	Prospective clinical	Grey Relational Analysis (GRA) + 4-layer DNN	150 valid clinical patients (stratified by age/gender)	Personalized shade selection	Integrating GRA-DNN achieves high clinical color matching accuracy for personalized shades.
41	Şahin et al. (2025) [43]	In vitro comparative study	In vitro	LLMs (ChatGPT-4, Gemini 1.5 Pro)	13 randomized acrylic resin teeth	Color matching	Spectrophotometers remain superior, but LLMs achieve an acceptable clinical color match.
42	Alqutaibi et al. (2025) [44]	Scoping review	N/A	Robotics, AI-assisted/guided robotics, automation	18 in vitro robotic studies analyzed	Tooth preparation/denture arrangement	Robotic prep arms show high mechanical precision but extended processing times.
43	Tay et al. (2026) [45]	Narrative Review	N/A	DCNNs, Transformers, XAI, NEMS	N/A	General dentistry/ prosthodontic relevance	Evaluates Explainable AI (XAI) transition; highlights need for validation and interoperability.
44	Vázquez-Sebrango et al. (2025) [46]	Systematic review	N/A	AI, ML, DL, CNNs, ANNs, SVM, RF, DT, KNN, LR, GANs	120 articles	Implant dentistry	Most current implant AI focuses on DL classification; performance depends on data quality, validation, and clinical integration.
45	Revilla-León et al. (2023) [47]	Systematic review	N/A	CNNs, SVR, k-NN, DTs	17 articles analyzed (JBI checklist applied)	Implant dentistry	AI-assisted implant planning reduces biomechanical stress compared with conventional approaches.
46	Wu et al. (2024) [48]	Scoping review	N/A	CNNs, Random Forests, SVMs, LR	12 studies analyzed	Implant prognosis	Deep learning predicts implant survival and prognosis, but models suffer from retrospective bias.
47	Danesh et al. (2023) [49]	Cross-Sectional Knowledge Assessment	Computational/Multiple-choice baseline	LLMs (ChatGPT-3.5 vs. ChatGPT-4)	143 board-style multiple-choice questions	Dental education	ChatGPT-4 outperforms ChatGPT-3.5 in clinical dental knowledge assessment.
48	Eraslan et al. (2025) [50]	Cross-Sectional Comparative study	Computational/MCQ benchmarking	LLMs (Copilot, Gemini Advanced, Claude Pro, ChatGPT-3.5, Perplexity)	126 prosthodontics questions	Prosthodontics education	AI chatbots show moderate accuracy across prosthodontic subtopics; implantology questions have the highest accuracy and removable partial dentures the lowest.
49	Freire et al. (2024) [51]	Cross-Sectional Accuracy Evaluation	Computational/Expert blind grading	ChatGPT-4	30 open-ended short questions (900 generated answers)	Prosthodontic Q & A/education	LLMs show limited clinical reliability in Q & A, with frequent plausible but incorrect answers.
50	Hooshiar (2025) [52]	Cross-Sectional Comparative Study	Computational	LLMs (GPT-4o, ChatGPT-4.1, ChatGPT-4.5 ScholarQA, OpenEvidence)	15 clinical implant questions (150 total answers)	Implant dentistry/LLM reliability	LLM outputs in implant dentistry are limited by factual inaccuracies and hallucinations, restricting clinical reliability.

Note: Highly detailed raw extraction data is available in the Supplementary File S2. N/A, not applicable.

**Table 3:** Summary of Artificial Intelligence Applications in Prosthodontics.

Application Domain	Main AI Role	Typical Techniques Reported	Main Advantages Reported	Main Limitations Reported
Diagnostic support and treatment planning	Detection, classification, decision support, and prognosis	- CNNs - Object detection algorithms (YOLOv3, YOLOv8, Mask R-CNN) - TransUNet and Swin-UNETR	- Standardizes diagnostic criteria across different setups and minimizes human errors - High accuracy (80–99%) for segmenting and identifying multi-specialty dental anomalies - Eliminates inter-observer variability	- Disconnect between laboratory performance and real-world clinical validation due to retrospective data bias - Panoramic-based bone models often disagree with clinical probing standards
Digital smile design and esthetic analysis	Facial landmark detection, esthetic simulation, and virtual smile planning	- Microsoft Face API (68 landmark engines) - GANs - Specialized DSD platforms (Smilecloud, REBEL)	- Predictable, facially guided planning achieved within fewer patient visits - Boosts patient engagement, visual communication, and final treatment acceptance - Comparable esthetic perception to manual design	- Standard AI templates struggle with facial asymmetry, leading clinicians to prefer human-guided manual designs on asymmetric profiles - Ethical concerns over algorithmic cultural bias and commercial over-treatment marketing.
Intraoral scanning and digital impressions	Real-time processing and alignment of raw digital data, automated soft-tissue artifact removal, and mesh-defect correction	- 3Shape Trios 5 AI Mesh-Filtering Engines - DL/MM architectures (Qwen-VL) - Confocal laser scanning and Active Wavefront Sampling	- Automatically removes unnecessary structural data (tongue, cheeks) to streamline digital workflows - More reliable complete-arch scanning by reducing arch distortion errors	- Automatic mesh filling must be monitored or deactivated on prepped margins due to accuracy changes - Performance may vary by scanner/system
Margin line detection/extraction	Automated segmentation and margin line extraction	- iMeshSegNet & Modified MeshSegNet - Point-cloud transformers (AdaPointTr) with Local Density + PCA - Semiautomated parametric line segmenters	- Reduces operator-dependent drawing variability and technical sensitivity. - More time-efficient margin line detection - Post-processing adjacency repair lowers invalid predictions by over 70% (DSC = 0.986)	- Semiautomated software options fail when convex and concave boundaries coexist along subgingival preparations - Highly sensitive to local surface steps or non-manifold mesh edges - Dataset dependence
CAD/CAM crown and prosthesis design	Automated design, morphology reconstruction, and prosthetic optimization	- StyleGAN, DCPR-GAN, and 3D-DCGAN - CrownSurfaceFormer (CSF) with Dimension Elevator Modules (DEMs) - Deformable FCN and Deformable Conv Modules	- Accelerates laboratory workflows, with reported time reductions of up to 52%. - High functional accuracy (P2P ≤ 0.66 mm) and superior internal fit (55.4 µm gap) - Improved morphology consistency	- Current AI libraries fail to reproduce real functional cusp wear facets, outputting idealized, unworn morphologies - Complex deep grooves/fossae suffer from mesh smoothing distortions
Color detection and shade matching	Color estimation, correction, and shade recommendation	- Extreme Learning Machines (ELMs) - Grey Relational Analysis (GRA) paired with 4-layer DNNs - MobileNetV2, ResNet18, Fuzzy Logic	- Lowers color differences (ΔE_00_) down to 1.0, matching strict clinical perceptibility criteria - Automatically integrates age/gender esthetic traits and limits ambient lighting shifts - More objective and reproducible shade selection	- Multimodal large language models show lower color-matching accuracy than spectrophotometers - Reflections from camera lenses and complex local tooth translucency can skew final outputs
Robotics in prosthodontics	Automated mechanical tooth preparations for crowns/veneers, complete denture arrangement assistance	- Single and multi-manipulator robotic arms - Ultra-Short Pulse Laser (USPL) photoablation end-effectors - 84-Degree-of-Freedom systems	- Delivers highly accurate, standardized tooth reduction profiles while reducing the effects of operator tremor and fatigue - Replaces time-consuming, operator-dependent handpiece setups	- Laser-guided cutting requires significant time (hours per crown) - High equipment and maintenance costs remain a major implementation barrier - Safety issues - Technical complexity - Limited routine use
Implant prosthodontics/implant dentistry	Mandibular nerve tracing, bone volume/density screening, brand classification, structural stress evaluation, planning, guided surgery, and prognosis	- ResNet-50, U-Net, YOLOv7, Swin-UNETR - Finite Element Analysis (FEA) integrated models - RPIFormer	- Yields over 95% accuracy in automatically identifying unknown implant brands on 2D radiographs - Minimizes localized stress patterns at the implant–bone interface.	- Prognostic and success prediction models suffer from high algorithmic bias due to retrospective dataset limitations - Fails to match higher-level clinical reasoning or complex long-term patient comorbidity traits
Education and chatbot/LLM applications	Assessment of dental knowledge, student-skill evaluation, virtual tutoring, and patient-facing informational support	- LLMs (ChatGPT-4, ChatGPT-4.5, Gemini Advanced, Claude Pro) - RAG platforms	- Useful as supplementary learning tools - Some models show moderate-to-good accuracy - RAG systems significantly reduce reference fabrication	- Conventional generative models frequently execute “hallucinations,” fabricating non-existent references, data, and statistics - Inconsistent reliability across prosthodontic topics - RAG architectures are highly resistant to reference fabrication but still risk text misinterpretations

## Data Availability

The data presented in this study are available in the article and Supplementary Materials. The standardized charting form and raw extraction datasets have been deposited in the Supplementary Materials.

## Supplementary Materials

The following supporting information can be downloaded at: https://www.mdpi.com/article/10.3390/dj14090570/s1, File S1: PRISMA-ScR checklist; File S2: Data Charting Form and Extraction Dataset.

## Abbreviations

The following abbreviations are used in this manuscript:

AI	Artificial Intelligence
PRISMA-ScR	Preferred Reporting Items for Systematic Reviews and Meta-Analyses Extension for Scoping Reviews
CNN/CNNs	Convolutional Neural Network/Convolutional Neural Networks
GAN/GANs	Generative Adversarial Network/Generative Adversarial Networks
CAD/CAM	Computer-Aided Design and Computer-Aided Manufacturing
DSD	Digital Smile Design
LLM/LLMs	Large Language Model/Large Language Models
XAI	Explainable AI (Explainable Artificial Intelligence)
ML	Machine Learning
DL	Deep Learning
ANN/ANNs	Artificial Neural Network/Artificial Neural Networks
JBI	Joanna Briggs Institute
N/A	Not Applicable
VLM	Vision-Language Model
MMs	Multimodal Models
R-CNN	Region-based Convolutional Neural Network
FDI	Fédération Dentaire Internationale
RPD/CD	Removable Partial Denture/Complete Denture
INN	Invertible Neural Network
SVM/SVMs	Support Vector Machine/Support Vector Machines
NNs	Neural Networks
Trans-VNet	Transformer-based Visual Network architecture
CV	Computer Vision
VR/AR	Virtual Reality/Augmented Reality
3D	Three-Dimensional
2D	Two-Dimensional
ICC	Intraclass Correlation Coefficient
API	Application Programming Interface
VDO	Vertical Dimension of Occlusion
ITU/WHO	International Telecommunication Union/World Health Organization
MeshSegNet	Mesh Segmentation Network
iMeshSegNet	Improved Mesh Segmentation Network
AdaPointTr	Adaptive Point Cloud Transformer
PCA	Principal Component Analysis
UQ	Uncertainty Quantification
DS	Digital Scan group/Cast Scans
IS	Intraoral Scan group
CDTs	Certified Dental Technicians
CSF	CrownSurfaceFormer
DEM	Dimension Elevator Modules
D-FCN	Dense Fully Convolutional Network
StyleGAN	Style-based Generative Adversarial Network
BPNN	Backpropagation Neural Network
YOLO	You Only Look Once (Object detection algorithm framework)
RF	Random Forest
XGBoost	Extreme Gradient Boosting
MobileNetV2	A lightweight convolutional neural network architecture
ResNet18	Residual Network with 18 layers
DenseNet121	Densely Connected Convolutional Network with 121 layers
EfficientNetB0	A highly optimized convolutional neural network baseline scaling model
ELM	Extreme Learning Machine
PR	Polynomial Regression
GRA	Grey Relational Analysis
DNN	Deep Neural Network
DCNNs	Deep Convolutional Neural Networks
Grad-CAM	Gradient-weighted Class Activation Mapping
NEMS	Nanoelectromechanical Systems
DT/DTs	Decision Tree/Decision Trees
KNN/k-NN	k-Nearest Neighbors
LR	Logistic Regression
FEA	Finite Element Analysis
SVR	Support Vector Regression
MCQ/MCQs	Multiple Choice Question/Multiple Choice Questions
RAG	Retrieval-Augmented Generation
Q & A	Question and Answer
TransUNet	Transformer-based U-Net architecture variant
Swin-UNETR	Swin Transformer for Medical Image Segmentation
4D	Four-Dimensional
IOS	Intraoral Scanner
DSC	Dice Similarity Coefficient
DCPR-GAN	Dental Crown Prosthesis Restoration Generative Adversarial Network
3D-DCGAN	3D Deep Convolutional Generative Adversarial Network
GRA-DNN	Grey Relational Analysis combined with a Deep Neural Network
RPIFormer	Removable Partial Implant Transformer
CBCT	Cone Beam Computed Tomography
